# Early-life adversity and long-term neurobehavioral outcomes: epigenome as a bridge?

**DOI:** 10.1186/s40246-017-0129-z

**Published:** 2017-12-16

**Authors:** Alexander M. Vaiserman, Alexander K. Koliada

**Affiliations:** Laboratory of Epigenetics, Institute of Gerontology, Vyshgorodskaya st. 67, Kiev, 04114 Ukraine

**Keywords:** Biological embedding, DNA methylation, Early-life adversity, Epigenetics, Neurobehavioral outcomes

## Abstract

Accumulating evidence suggests that adversities at critical periods in early life, both pre- and postnatal, can lead to neuroendocrine perturbations, including hypothalamic-pituitary-adrenal axis dysregulation and inflammation persisting up to adulthood. This process, commonly referred to as biological embedding, may cause abnormal cognitive and behavioral functioning, including impaired learning, memory, and depressive- and anxiety-like behaviors, as well as neuropsychiatric outcomes in later life. Currently, the regulation of gene activity by epigenetic mechanisms is suggested to be a key player in mediating the link between adverse early-life events and adult neurobehavioral outcomes. Role of particular genes, including those encoding glucocorticoid receptor, brain-derived neurotrophic factor, as well as arginine vasopressin and corticotropin-releasing factor, has been demonstrated in triggering early adversity-associated pathological conditions. This review is focused on the results from human studies highlighting the causal role of epigenetic mechanisms in mediating the link between the adversity during early development, from prenatal stages through infancy, and adult neuropsychiatric outcomes. The modulation of epigenetic pathways involved in biological embedding may provide promising direction toward novel therapeutic strategies against neurological and cognitive dysfunctions in adult life.

## Background

A growing body of research in recent years highlights the importance of early-life environmental influences in determining the adult health status. On the base of these findings, the Developmental Origin of Adult Health and Disease (DOHaD) hypothesis was proposed postulating that unfavorable environmental early-life conditions can result in “developmental programming” of later-life chronic disease [1, 2]. This hypothesis has been initially focused on the lifelong outcomes of prenatal and neonatal malnutrition. In recent years, however, it became increasingly apparent that non-nutritional impacts such as psychological stress exposure during development can also greatly affect the health status throughout adult life, and epigenetic regulation is considered as a key mechanism mediating these effects.

Most research evidence for the developmental programming by stressful conditions early in life is obtained in rodent models (for reviews, see references [3–6]). In these studies, convincing evidence has been obtained to indicate that early stressful exposures such as perinatal stresses, maternal separation, and inadequate maternal care can cause marked neuroendocrine perturbations persisting up to adulthood and causing impaired cognitive, behavioral, and social functioning during the adult life. A body of studies has demonstrated that prenatal stress and exposure to excess levels of exogenous glucocorticoids can both be related to unfavorable health outcomes including low birth weight, neuroendocrine pathology, and enhanced risk for cardio-metabolic, infectious, and psychiatric disorders throughout the adult life [7–9]. The neuroendocrine effects triggered by prenatal stress have been reported to be associated with depressive- or anxiety-like behavioral phenotypes, including altered levels of physical activity, enhanced immobility throughout a forced swim test, and lowered exploration of novel environments [10]. These effects were shown to be mediated by changes in both maternal and fetal hypothalamic-pituitary-adrenal (HPA) axes causing intrauterine exposure to glucocorticoid excess [11, 12]. A role for in-utero glucocorticoid exposure induced by maternal stress in rats is evident from research in adult offspring born to either mothers with an intact corticosterone secretion or to intrauterine-stressed adrenalectomized dams [13]. The maternal stress-induced glucocorticoids can pass the placental barrier and thereby disrupt the development of the fetal brain. The stress-related maternal-placental-fetal endocrine and immune/inflammatory candidate mechanisms were proposed as possible candidate mechanisms for long-term effects of the fetal stress exposures on physiological characteristics of the developing organism [14, 15]. Early postnatal stages are another important sensitive period for developmental programming. In rodent research, it was shown that impaired mother-infant interactions (e.g., maternal deprivation/separation) throughout the postnatal period can substantially impair the neuroendocrine regulation, including upregulation of hippocampal glucocorticoid receptor (GR) and hypothalamic corticotropin-releasing factor (CRF), and also can lead to enhancement of the adrenocorticotropic hormone and corticosterone levels [16–18]. Such early adversity-induced neuroendocrine changes may lead to behavioral issues during adulthood, including the impaired learning, memory, and also depressive- and anxiety-like behaviors [19]. The long-term effects caused by variation in postnatal maternal care in rodents (e.g., low or high levels of licking and grooming, LG) are most studied in this context to date. The offspring of high-LG mothers exhibited lower levels of stress responsivity, better performance on cognitive tasks, and exploratory behavior in a novel environment during adulthood than the offspring that have been reared by low-LG dams [20, 21]. In these studies, the physiological and biochemical changes induced by adversities in early life were accompanied by substantial alterations on the level of epigenetic control of gene expression and related changes in patterns of DNA methylation, histone modifications, and microRNA regulation.

The evidence for long-lasting effects of adversities in early life from human studies are rather scarce and mainly limited to change in DNA methylation level which is thought to be the most stable form of epigenetic modification. This review is mostly focused on the results obtained from human studies highlighting the causative role of epigenetic pathways in mediating the link between the adversity in early development from prenatal stages through infancy and adult neuropsychiatric outcomes.

## Search strategy

In this review, we searched the PubMed database (http://www.ncbi.nlm.nih.gov/pubmed/) to find all published studies on the epigenetic links (both at the genome-wide and candidate gene levels) between early-life adversity and long-term neurobehavioral outcomes in humans. In our search, we used combinations of the following search terms: “biological embedding,” “early-life adversity,” “epigenetic,” “epigenome,” “DNA methylation,” “neurobehavioral,” and “neuropsychiatric.” The time period of the search covered articles published from 1994 to 2017 with no language restrictions, although only English language studies were eventually included. There was no restriction on the type of study design; therefore, all clinical, epidemiological, and quasi-experimental studies satisfying the search criteria were included. Several relevant experimental studies closely related to the topic under discussion were also eligible for inclusion. We used these papers to determine whether there is a coherence of effects across humans and non-human species and to examine the contribution of epigenetic mechanisms in biological embedding of adverse early-life exposures.

## Biological embedding of adverse experiences in early life

Currently, the regulation of gene activity by epigenetic mechanisms (mitotically or meiotically heritable changes in gene expression that occur without any change in DNA sequence) is suggested to be a key player in mediating the link between stressful events early in life and adult neurobehavioral outcomes [22, 23]. DNA is known to maintain stability during the whole life cycle (except for mutations that occur randomly), while the epigenetic marks are dramatically changed throughout the early developmental stages to initiate distinguished patterns of expression among different developing tissues. The main mechanisms of epigenetic regulation in mammals are covalent modification of DNA by methylation, post-translational modifications (including acetylation, phosphorylation, methylation, and ubiquitination) of the histone proteins, as well as regulation by non-coding RNAs (ncRNAs) [24].

There are numerous lines of evidence indicating that the mammalian epigenome (i.e., the totality of epigenetic marks across the whole genome) is the most labile and, thereby, most sensitive to various environmental and hormonal cues, at specific stages of early development [25]. In mammals, a global demethylation of DNA followed by remethylation was shown to occur throughout the development of germ cells. A second genome-wide demethylation wave takes place in early embryogenesis, and patterns of methylation are re-established after implantation of the blastocyst [26]. The phases of post-fertilization demethylation and remethylation are likely playing a role in the removal of epigenetic information acquired by the parental generation [27, 28]. Once established throughout early development, epigenetic marks are stable maintained through cell division.

The epigenome thereby seems particularly susceptible to unfavorable environmental conditions during the stages of gametogenesis and early embryogenesis [29]. In mammals, including humans, the period of maximal epigenetic plasticity continues from before birth until weaning [30]. Various environmental cues in early life, particularly severe stresses or trauma, can cause lifelong epigenetic modifications which may, in turn, set the organism off on phenotypic trajectories to health or disease [31, 32]. There are numerous evidences from animal studies that environmental adversities and/or psychosocial stresses early in life can trigger epigenetic modifications with significant functional consequences for brain plasticity and behavior and subsequently lead to a variety of cognitive dysfunctions and psychiatric disorders in adult life [33–36]. The crucial role of epigenetic machinery in the biological embedding of stressful exposures in early life has been demonstrated in a number of rodent models, where considerable variations in both DNA methylation and histone modification have been reported in offspring exposed to different prenatal stresses, inappropriate maternal care, maternal deprivation/separation, as well as to juvenile social enrichment/isolation [3–6].

Remarkably, the earlier the organism is affected by stressful experiences during the intrauterine period, the more pronounced long-term consequences are usually observed, suggesting a causative role of epigenetic processes in pathways to adult-life pathological conditions. One good example for that is a mouse study by Mueller and Bale [37], where exposure to various stressful events during fetal development resulted in elevated stress sensitivity in adulthood, which has been manifested in modified expression of GR and corticotropin-releasing hormone, and also in the enhanced responsivity of HPA axis in prenatally affected animals. These effects have been accompanied by changes in methylation levels and in expression of GR and CRF genes. The period of early gestation was identified in this study as a particularly sensitive stage, suggesting a strong evidence for epigenetic involvement in developmental programming of neuroendocrine functions.

Postnatal exposures, such as neonatal handling (an experimental procedure in which animals are briefly separated from the dam and handled for the first 10 postnatal days), were also shown to have a profound impact on epigenetic profiles. This is a widespread experimental procedure used to understand how adversity early in life can affect neurobehavioral development of animals and place them on a pathway to disease. It has been found that neonatal handling can induce a persistent increase in the transcription level of the nuclear receptor subfamily 3, group C, member 1 (NR3C1) gene encoding the GR [38]. The epigenetic changes, such as those triggered by handling, have been observed in offspring raised by high-LG mothers. Among them, there was a reduced level of DNA methylation in the promoter region of the hippocampal NR3C1 gene [39]. These effects occurred throughout the first week of postnatal life, and they have been shown to be reversed by cross-fostering, persisted into adulthood, and associated with changed histone acetylation and transcription factor (NGFI-A) binding to the GR promoter. Another gene that was found to be expressed in the adult prefrontal cortex in rats in response to an adversity in early life is the brain-derived neurotrophic factor (BDNF) gene playing a crucial role in the neural and behavioral plasticity and in development of various psychiatric disorders related to adversity early in life, such as depression, bipolar disorder, autism, and schizophrenia [40]. These expression changes have been accompanied by corresponding changes in the methylation levels.

Findings from human studies suggesting a role of epigenetic mechanisms in long-lasting effects of adversities in early life are more limited compared to those obtained from animal models due to the restricted access to suitable biological materials, but they clearly demonstrate that these mechanisms can also operate in man. In the succeeding subsections, findings from human studies are summarized and discussed.

### Maternal adversity in pregnancy

It has been well documented in many human studies that maternal exposure to adverse conditions during pregnancy, including an unfavorable social environment, anxiety, depression, starvation, and pain (all known to increase the intrauterine level of glucocorticoids), can be linked to a variety of cognitive and behavioral problems during the adulthood [41–43]. There is consistent evidence that antenatal stress or anxiety has a programming effect on the fetus which can persist up to adulthood and result in an elevated risk of psychiatric and behavioral pathological conditions, including autism, schizophrenia and anxiety/depression-related behaviors, and also impaired cognitive performance in later life [44].

Recent studies highlighted the role of epigenetic mechanisms in mediating long-lasting outcomes of maternal adversities during pregnancy. Pre- and early postnatal dysregulation of epigenetic pathways resulted in genome-wide modulating gene expression in different tissues including the brain, by that influencing the functioning and connectivity of neural circuitry and affecting the risk for neurobehavioral impairments in later life [41]. In a methylome-wide association study (MWAS), maternal depression-associated changes in the DNA methylation levels were revealed in neonatal T lymphocytes; these alterations were found to persist to adult age in the hippocampal tissues [45]. A strong association was observed between maternal depressive symptoms during pregnancy and increased level of *NR3C1* exon *1F* methylation in male infants, and also lowered methylation level of another gene responsible for these associations, *BDNF IV*, in both male and female infants [46]. Gestational exposure to maternal depressed or anxious mood in the third trimester of prenatal development caused an enhanced methylation in the CpG-rich region of the promoter and exon1F of the GR gene (NR3C1) in the newborn cord blood, and these effects have been demonstrated to be persistent throughout the infancy [47]. Surprisingly, these epigenetic effects were revealed in offspring but not in maternal blood samples. The methylation levels of NR3C1 gene in cord blood have been associated with the levels of stress response in 3-month-old infants (as measured by salivary cortisol levels), assuming functional consequences of these epigenetic variations for the HPA stress responsiveness. Radtke et al. [48] revealed that maternal exposure to intimate partner violence throughout pregnancy affected the methylation level of NR3C1 gene in the whole blood DNA of 10–19-year-old adolescent offspring. As in the case of the maternal depression during pregnancy, these epigenetic effects were observed in affected offspring, but not in maternal blood. The same effects have been seen in cord blood as a result of maternal pregnancy-associated anxiety [49]. Similar findings were also reported on the SLC6A4 gene encoding the serotonin transporter. The methylation levels of SLC6A4 have been shown to be associated with a number of prenatal and postnatal adverse exposures, including maternal depression during pregnancy, as well as childhood trauma and abuse [50]. Prenatal exposure to a maternal depressed mood throughout the 2nd trimester of gestation has been revealed to be associated with a decreased methylation level in promoter region of SLC6A4 gene, in leukocytes from maternal peripheral blood and in umbilical cord leukocytes obtained from neonates at birth, while no such effects regarding the BDNF gene were observed [51].

In several studies, importance of epigenetic regulation of placental genes, playing a crucial role in maternal-fetal interactions, in long-lasting outcomes of maternal adversity has been reported. Prenatal exposure to maternal anxiety and/or depression has been shown to adversely influence the neurobehavioral development of newborns. These unfavorable neurodevelopmental outcomes have been demonstrated to be linked to the increased methylation levels of the NR3C1 and 11β-hydroxysteroid dehydrogenase type 2 (11β-HSD2) placental genes and to significant perturbations of the HPA axis [52]. The expression levels of the placental human SLC6A4 gene were found to be substantially elevated in placentas from mothers who had untreated mood disorders during pregnancy in comparison with control women [53]. An association between the maternal mood throughout the pregnancy and downregulation of placental 11β-HSD2 gene encoding the cortisol-metabolizing enzyme was revealed in the study by O’Donnell et al. [54]. The infants whose mothers were exposed to higher socioeconomic adversity levels in their pregnancy have also been found to have the lowest methylation levels in the placental 11β-HSD2 gene [55]. The authors have suggested that such methylation patterns of this gene indicate that cues from environment transmitted from mother to fetus during gestation may program the response to potentially unfavorable environment in postnatal life via lesser exposure to cortisol throughout prenatal development. Overall, the findings from these investigations indicate that placental genes can be implicated in intrauterine programming of neurological functioning.

**Fig. 1:**
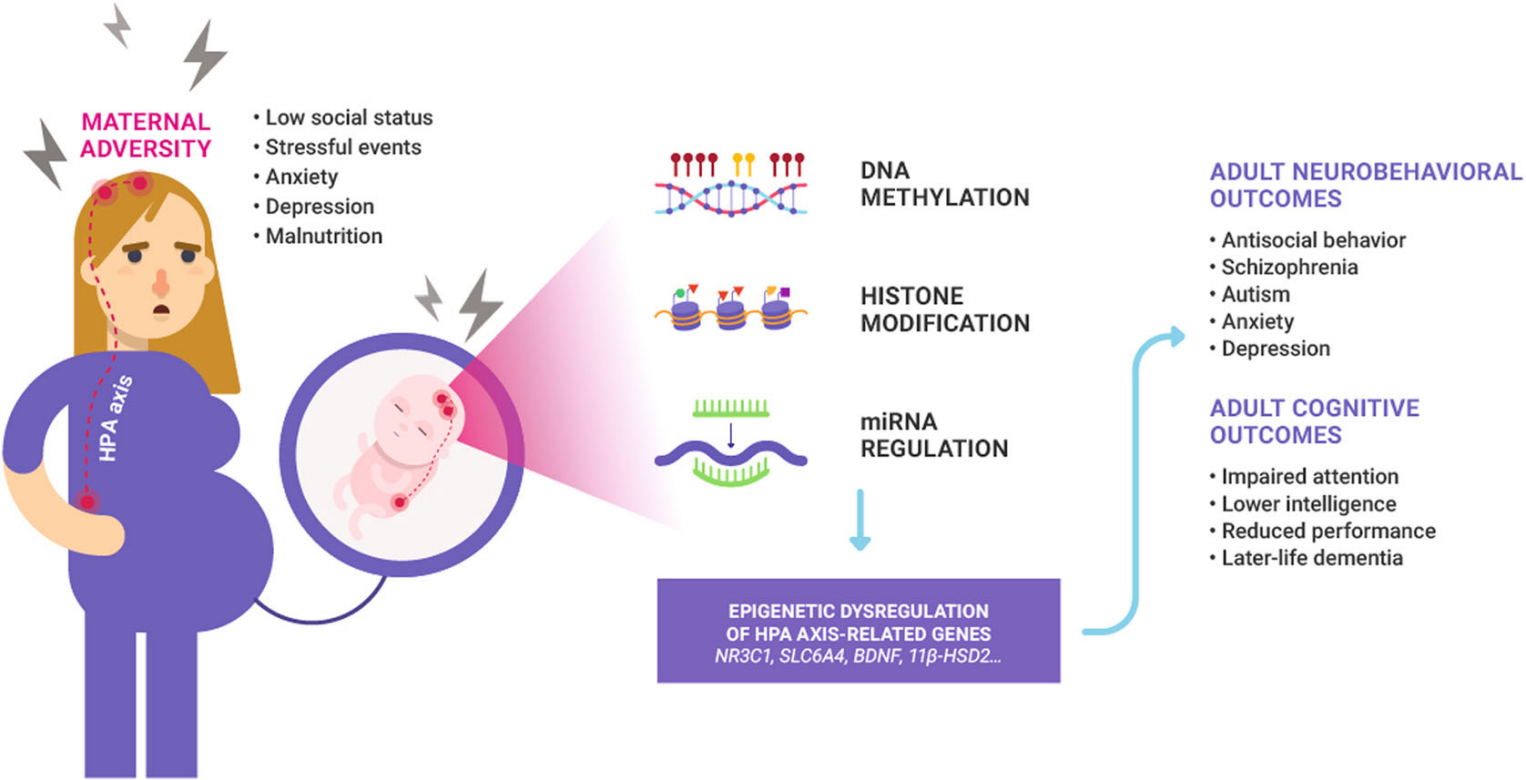
Schematic representation of hypothetical mechanisms linking maternal adversity in pregnancy to neurobehavioral and cognitive dysfunction in offspring

A schematic representation of hypothetical mechanisms linking maternal adversity in pregnancy to neurobehavioral and cognitive dysfunction in offspring is given in Fig. 1.

### Adversity in childhood

In addition to in-utero developmental stage, early infancy is another critical stage of epigenetic plasticity. Early postnatal development is characterized by very rapid growth of various organs and organ systems including the brain and the rest of the nervous system, and epigenetic regulation is regarded as a crucial process through which the formation of specific synapses occurs throughout critical developmental periods [56, 57]. Therefore, in addition to adversities throughout the gestational development, early postnatal adversity can also have a potential for long-term epigenetic programming.

There is increasing evidence that unfavorable conditions in early infancy related to maltreatment, poor quality parenting or loss of parents, parental psychiatric disorders, exposures to physical, sexual, psychological, or emotional abuse, etc. can lead a number of adverse neurobehavioral and cognitive outcomes in adulthood [58–60]. Since the central nervous system interacts with the immune system via the HPA axis and autonomic nervous system (ANS), the immune dysregulation is regarded as a core component of these programming effects. Adversity in early life has been shown to be related to alterations in neural development (particularly of the hippocampus, amygdala, and prefrontal cortex), ANS and HPA axis dysregulation [61], and enhanced levels of inflammatory mediators [62]. The impaired neural development is believed to be a central pathway by which adversity early in life can increase the inflammation level and thereby the risk for adverse psychophysical health outcomes. Moreover, the exposure to chronic stressful conditions in infancy can lead to failure or depletion of normal physiologic processes (“allostatic load hypothesis”) and thereby impair the physiological response to stress and other health outcomes in adult life through a process called biological embedding [62–65]. Adult subjects having a history of adversity in their childhood showed the decreased volumes of prefrontal cortex and hippocampus, enhanced level of activation of HPA axis in response to stress, and elevated inflammation levels compared to non-maltreated persons [63].

Long-lasting emotional and cognitive dysfunctions caused by adversities in early life are thoroughly studied in rodent models. Typically, animals exposed to a postnatal maternal deprivation demonstrate elevated neuroendocrine response to stress, cognitive impairment, and enhanced levels of anxiety and depressive-like behavior [66–68]. Several phenotypes reported in these models of early-life adversities were likely to share common neurobiological mechanisms. So, there is evidence for impaired glucocorticoid negative-feedback control of the HPA axis, reduced hippocampal neurogenesis, and altered glutamate neurotransmission in both prenatally stressed rats and those animals that experienced inadequate maternal care [68].

These findings from animal models have been extended to humans by highlighting associations between adversities in early life and modified epigenetic patterns in adulthood [69–72]. Substantial epigenetic effects were revealed for the assortment of genes involved in the etiology of conversion disorders, aggressive and suicidal behaviors, and callous-unemotional traits [73, 74]. Among these genes, most are involved in mediating the HPA axis, brain development, immune response, neurotransmission, serotonin synthesis, and other processes. In some studies, psycho-emotional trauma in childhood has been demonstrated to be a potential risk factor for developing depressive symptoms later in life, particularly in response to additional trigger stressful events [75, 76]. For instance, women having a history of childhood abuse and actual diagnosis of major depression showed a sixfold higher level of adrenocorticotropic hormone stress response than the age-matched control individuals, suggesting that there may be permanent changes in set-points for HPA activity in response to stress among those persons who were exposed to early-life stressful conditions [77]. On the basis of these findings, Heim et al. suggested that trauma early in life is related to the sensitization of neuroendocrine stress responses, immune activation, enhanced central CRF activity, glucocorticoid resistance, and lower hippocampal volume throughout the adult life [75]. These neuroendocrine changes triggered by stresses in early life can likely affect the risk of developing depression in response to stress during adulthood. Data from recent studies highlight the critical role of epigenetic regulation in the linkage between trauma in childhood and depression in adult life [76].

It should be noted, however, that the link between adverse conditions in early life and unfavorable neurobehavioral outcomes in adulthood can be dependent not only on epigenetic processes per se, but also on the genetic background of affected individuals. In particular, different combinations of functional polymorphisms in dopamine and serotonin pathway genes can result in both responder and non-responder phenotypes in the wide range from adverse to advantageous early-life circumstances. For example, a functional polymorphism in the promoter gene of monoamine oxidase A (MAOA), a mitochondrial enzyme that degrades the neurotransmitters including serotonin, norepinephrine, and dopamine, was demonstrated to mediate the association between adversities early in life and enhanced risk for violence and antisocial behavior in adulthood. In the research by Frazzetto et al., the MAOA genotype was found to moderate the link between traumatic events experienced from birth up to the age of 15 years and physical aggression in adult life, as assessed by the Aggression Questionnaire [78]. In this study, scores of physical aggression were shown to be higher in those adult men who have been exposed to traumatic events early in life and who carried the low MAOA activity allele (MAOA-L). These findings were confirmed by later studies. The interaction of MAOA genotype and childhood adversity on antisocial outcomes was examined in a meta-analysis of 27 studies conducted by Byrd and Manuck [79]. Across 20 male cohorts, adversity in early life has been demonstrated to be a stronger predictor of adult antisocial outcomes for a low-activity, compared to a high-activity, MAOA genotype. Similar, but less consistent, findings were reported in 11 female cohorts studied.

An association between the adversities early in life and long-lasting changes in processes of epigenetic regulation at the whole-genome level was demonstrated repeatedly. Many of such studies used low socioeconomic status (SES) as indicator of early stressful conditions. Low SES, generally accompanied by an enhanced stress load due to a poor quality of nutrimental intake, infections, and higher load of physical work, has been found to strongly predict a number of psycho-emotional pathologies such as schizophrenia and depression in adult life [67]. Disadvantaged early SES was related to profiles of adult blood DNA methylation [80]. Most of the genes differentially methylated in association with low early-life SES are known to be functionally implicated in metabolic and cell signaling pathways. Genome-wide transcriptional profiling demonstrated that in healthy adults with low-SES childhood background, genes bearing response elements for CREB/ATF family of transcription factors transmitting adrenergic signals to leukocytes were substantially upregulated, whereas genes with response elements for the GR, regulating the secretion of cortisol and transducing the anti-inflammatory signals to the immune system, were significantly downregulated [81]. Individuals exposed to low-SES conditions in early life also exhibited raised cortisol levels, elevated expression of pro-inflammatory transcription factor NF-kappaB, as well as increased production of the pro-inflammatory cytokine interleukin 6. On the basis of data obtained, the authors suggested that “low early-life SES programs a defensive phenotype characterized by resistance to glucocorticoid signaling, which in turn facilitates exaggerated adrenocortical and inflammatory responses. Although these response patterns could serve adaptive functions during acute threats to well-being, over the long term, they might exact an allostatic toll on the body that ultimately contributes to the chronic diseases of aging.” Chen et al. have revealed that the unfavorable effects of the low-SES conditions in early life on immune system functioning and inflammatory processes in adult life can be at least partly prevented by the high-level maternal warmth [82]. These alterations have been accompanied by changes in genome-wide transcription profiles. Those individuals who had low SES level early in life and whose mothers demonstrated high warmth toward them showed reduced Toll-like receptor-stimulated production of interleukin 6 and lowered activity of immune activating transcription factor (AP-1) and NF-kappaB in comparison with those subjects who had low SES level early in life but have experienced lower maternal warmth. These findings suggest that disadvantageous effect of low socioeconomic environment in early life might be buffered by a supportive family climate.

Similar lasting effects have been obtained for the unfavorable experiences such as sexual/physical abuse or neglect in early life [83]. Three hundred sixty-two differentially methylated promoters have been identified by a genome-wide analysis in the hippocampal neurons isolated from postmortem brain samples in subjects with a history of heavy abuse throughout infancy in comparison with control persons [84]. Among them, those genes implicated in a cellular or neural plasticity were shown to be the most differentially methylated. Nine hundred ninety-seven gene promoters were identified as being differentially methylated in association with abuse throughout childhood in a whole-blood DNA from adult subjects [85]. Most of these genes are involved in important pathways of cellular signaling associated with development and regulation of transcription. Four hundred forty-eight gene promoters were differentially methylated in T cells from adult male individuals exposed to parental physical aggression in the age of 6 to 15 years relative to a control group [86]. Most these genes are known to play an important role in aggressive behavior.

Long-lasting social and behavioral problems were also observed in persons who experienced parental neglect through the institutionalization in early life [87]. Differential patterns of whole-genome DNA methylation were found in blood samples from institutionalized children and children reared by their biological parents [88]. Most of these differentially methylated genes are related to immune and cellular signaling pathways, including those responsible for development and functioning of the brain as well as in neural communication. One hundred seventy-three genes were differentially methylated among subjects with and without the placement into the foster care system during their childhood [69]. Most of these genes are involved in the ubiquitin-mediated proteolysis pathway, which plays an important role in immune/inflammatory responses, in antigen processing and presentation pathways, and also in some important cellular processes. Moreover, 72 genes known to be related to the control of apoptosis and transcriptional regulation exhibited increased methylation levels in those individuals who had a history of foster care placement, while 101 genes involved in protein catabolic processes and in the control of posttranslational protein modification exhibited lowered levels of methylation in comparison with control subjects. Summary of evidence on the epigenetic link between adverse early-life events and adult-life neurobehavioral outcomes obtained from epigenome-wide association studies (EWAS) is given in Table 1. As we can see from Table 1, most of these EWAS data have been obtained from small samples; therefore, one must use some caution in interpreting these results. Further studies with larger samples are clearly required in order to allow more reliable conclusions.

**Table 1:** Summary of evidence on the link between adverse early-life events and adult neurobehavioral outcomes from epigenome-wide association studies

Condition /exposure	Stage at exposure	Age at detection	Tissue/cells	Population, sample size (*n*)	DMRs or up/downregulated genes, *n*	Function/pathway	Ref.
Maternal depression	Prenatal	Adult	Hippocampal tissue samples	Male postmortem samples with (*n* = 12) or without (*n* = 50) a history of maternal depression	294 DMRs associated with 234 genes	Immune system functions	[45]
Low SES	Childhood	45 years	Blood	40 British adults	586 hypermethylated and 666 hypomethylated gene promoters	Cell signaling pathways	[80]
	Childhood	25–40 years	Blood	103 healthy adults	73 upregulated and 37 downregulated genes	Raised cortisol levels; increased IL-6 production	[81]
	Childhood	25–40 years	Blood	53 healthy adults with a history of low early-life SES	330 upregulated and 161 downregulated genes in participants who grew up with high maternal warmth	Immune activation and systemic inflammation; diminishing these outcomes by supportive family climate	[82]
Child neglect/abuse	Childhood	Adult	Hippocampal neurons	25 French-Canadian men with a history of severe childhood abuse and 16 control subjects	248 hypermethylated DMRs; 114 hypomethylated DMRs	Cellular/neuronal plasticity	[84]
	Childhood	45 years	Blood	12 British men with a history of childhood abuse and 28 control subjects	311 hypermethylated and 686 hypomethylated gene promoters	Development, regulation of transcription	[85]
	Childhood	Adult	T lymphocytes	8 subjects with a history of physical aggression from age 6 to 15 years and 57 controls	171 hypermethylated and 277 hypomethylated gene promoters	Aggressive behavior	[86]

*DMRs*, differentially methylated regions; *SES*, socioeconomic status

In addition to EWAS, consistent evidence for the importance of epigenetic regulation in mediating long-term effects of early adversity was also provided from some candidate gene research. While a full-genome analysis allows to generate hypotheses on the underlying molecular mechanisms, the candidate gene approach allows to determine whether a specific gene of interest makes a contribution in each particular case. For example, in the McGowan et al. study, epigenetic differences in the brain loci substantially involved in the pathophysiology of suicide have been observed [89]. Specifically, by studying the postmortem hippocampal brain samples from suicidal individuals, the history of neglect/abuse in early childhood was shown to be associated with a lowered hippocampal volume and with severe cognitive impairments. Moreover, the gene encoding ribosomal RNA (rRNA) was significantly hypermethylated throughout the promoter and 5′ regulatory region in the brains of suicide victims, consistent with the decreased level of expression of rRNA gene in the hippocampus. Subsequently, McGowan et al. [90] examined epigenetic differences in a neuron-specific promoter of NR3C1 gene among postmortem hippocampal tissues from suicide completers with or without the child abuse history. The NR3C1 gene was selected for analysis since the decreased level of GRs within the hippocampus is believed to lead to elevated HPA stress response and thereby might account for an enhanced risk of psychopathology and poorer emotional regulation in those subjects who were abused in childhood. In that study, the levels of expression of NR3C1 gene were considerably decreased in suicide victims having a history of childhood abuse compared to non-abused suicide victims or control individuals; no differences, however, were revealed among non-abused suicide victims and control subjects. The essential effect on the expression of transcripts from the exon 1F NR3C1 promoter has been also observed. Labonté et al. also reported the enhanced methylation levels in the promoter of the 1F NR3C1 and decreased expression of this gene in the hippocampus of suicide completers having a history of abuse compared to either suicide completers with no abuse history or to control subjects [91]. In a more recent research by Bustamante et al., the childhood maltreatment assessed by a retrospective self-report questionnaire has been significantly associated with methylation levels in the NR3C1 promoter region in whole blood of adult persons [92]. Tyrka et al. also demonstrated that adversity in childhood can be linked to the risk for adult psychiatric disorders via epigenetic regulation of glucocorticoid signaling genes such as NR3C1 and gene coding for FK506 binding protein 51 (FKBP5) [93]. In another recent study by the same authors, the reduced methylation levels of NR3C1 gene were significantly associated with maltreatment in childhood and anxiety, depressive and substance-use disorders in adulthood [94]. An enhanced risk for development of stress-associated psychiatric disorders in adulthood was shown to be associated with childhood trauma-dependent, allele-specific demethylation of the functional glucocorticoid response elements of FKBP5 gene playing an important role in regulation of the HPA axis [95]. The demethylation of FKBP5 gene has been associated with elevated stress-dependent gene transcription followed by lasting dysregulation of the stress hormone system and by global effect on immune functioning and areas of the brain related to stress regulation.

One important issue in these studies is limited access to human neural tissues. Thereby, most candidate gene studies examining role of epigenetic variations in developmental programming of adult behavioral and cognitive dysfunctions are based on samples from peripheral tissues. In the above-mentioned study by Bick et al., significant negative correlation was observed between the mothers’ parenting reports and methylation levels of NR3C1 gene and also a macrophage migration inhibitory factor gene functionally implicated in the expression of NR3C1 and immune response in offspring blood 5 to 10 years after assessing the maternal caregiving quality [69].

Childhood-adversity caused epigenetic modifications in NR3C1 gene in the human blood samples have also been reported in the Tyrka et al. research [96]. In this study, enhanced levels of NR3C1 methylation were observed in leukocyte DNA from healthy adult individuals exposed to inadequate nurturing or maltreatment during their childhood. The elevated methylation levels of the exon 1F NR3C1 promoter in the peripheral blood of individuals suffering from borderline personality disorder or major depressive disorder have been revealed to be associated with the severity of childhood maltreatment [97]. No such changes have been found, however, in bulimic women exposed to abuse in their childhood [98]. Similar findings have been obtained for a serotonin system playing a crucial role in the brain development (including a region important in stress-regulation such as the hippocampus) and in the etiology of depression [99]. In this study, the childhood trauma, along with male gender and smaller hippocampal volume, has been independently associated with higher levels of peripheral serotonin transporter methylation in adulthood.

In some candidate gene researches, low SES has been used as a reliable indicator of adverse early-life conditions. In the research by Miller and Chen, the adolescent subjects whose families owned homes throughout their early childhood demonstrated higher levels of NR3C1 expression and lower levels of toll-like receptor 4 (TLR4) gene expression in leukocytes from peripheral blood compared to individuals with low SES in early life [100]. Data from this study indicated that low SES early in life may trigger a pro-inflammatory phenotype in later life. Similar findings have been obtained in African-American men who often have low SES in their childhood. In the study by Witek-Janusek et al., higher levels of childhood trauma and indirect exposure to neighborhood violence have been shown to be related to a greater acute stress-induced IL-6 response and also to a reduced methylation of the IL-6 promoter and lower cortisol response in adulthood [101]. Summary of evidence on the epigenetic link between adverse early-life events and adult-life neurobehavioral outcomes obtained from candidate gene studies is given in Table 2.

**Table 2:** Summary of evidence on the epigenetic link between adverse early-life events and adult neurobehavioral outcomes from candidate gene studies

Condition/exposure	Stage at exposure	Age at detection	Tissue/cells	Population, sample size (*n*)	Function/pathway	Gene/element	Epigenetic outcome	Ref.
Low SES	0–5 years	25–40 years	Saliva	103 adults	Decreased glucocorticoid and increased pro-inflammatory signaling	*CREB/ATF* gene family *NR3C1*	Upregulation Downregulation	[81]
Child maltreatment	Early childhood	Adulthood	Postmortem hippocampus	18 male suicide subjects, 12 controls	Impaired ribosomal functioning	*RRNa* promoter	Hypermethylation	[89]
	Childhood	Adulthood	Postmortem hippocampus	12 abused and 12 non-abused suicide subjects, 12 controls	Impaired stress reactivity	*NR3C1*	Downregulation	[90]
	Childhood	Adulthood	Postmortem hippocampus	21 abused and 21 non-abused suicide subjects, 14 controls	Impaired stress reactivity	*NR3C1 1F*	Hypermethylation	[91]
	Childhood	Adulthood	Whole blood	74 maltreated and 73 control subjects	Impaired stress reactivity	*NR3C1 1F* promoter region	Hypermethylation	[92]
	Childhood	18–59 years	Leukocytes	58 female and 41 male subjects	Impaired stress reactivity	*NR3C1* promoter	Hypermethylation	[93]
	Childhood	18–65 years	Leukocytes	213 female and 127 male subjects	Impaired stress reactivity	*NR3C1* promoter	Hypomethylation	[94]
	Childhood	19–59 years	Peripheral blood cells	30 subjects with and 46 without the history of child trauma	Immune functioning, stress reactivity	Glucocorticoid response elements of *FKBP5* gene	Allele-specific demethylation	[95]
	Childhood	18–59 years	Leukocytes	58 female and 41 male subjects	Impaired stress reactivity	*NR3C1* promoter	Hypermethylation	[96]
	Childhood	Adulthood	Peripheral blood	200 subjects with different rate of child maltreatment	Impaired stress reactivity	*NR3C1 1F* promoter	Hypermethylation	[97]
	Childhood	18–65 years	Peripheral blood	33 maltreated subjects, 36 controls	Stress-related psychopathology	Serotonin transporter gene promoter	Hypermethylation	[99]
	Childhood	18–25 years	Peripheral blood	34 African American men	Higher proinflammatory response to stress	*IL-6* gene promoter	Hypomethylation	[101]

Overall, these research findings highlight the mechanisms implicated in early adversity-induced impairment of neuroendocrine pathways associated with stress reactivity and adult social behavior. One of the most significant health outcomes of the childhood adversity is lasting neuroendocrine disturbance caused by adversity-induced alterations in the methylation levels of NR3C1 gene, thereby leading to changed cortisol production and various pathological conditions in adulthood [39, 102].

**Fig. 2:**
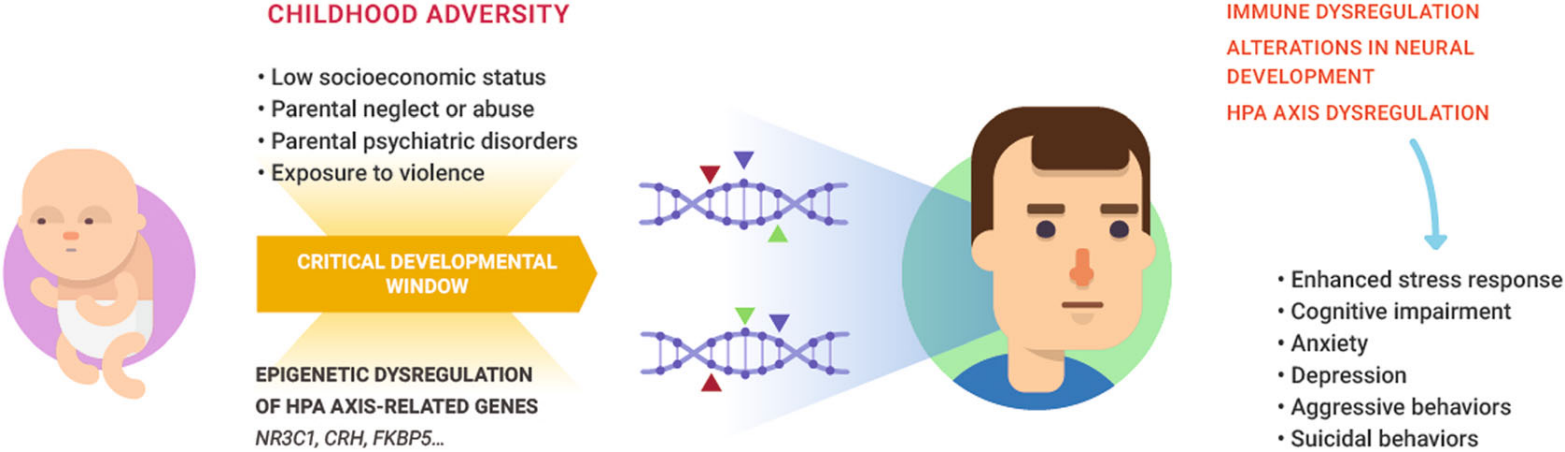
Schematic representation of hypothetical mechanisms linking childhood adversity to later-life neurobehavioral and cognitive dysfunction

A schematic representation of hypothetical mechanisms linking childhood adversity to later-life neurobehavioral and cognitive dysfunction is given in Fig. 2.

### Natural experiment-based evidence

Causal relationship between stressful events in early life and health problems later in life is evident from a body of quasi-experimental states (“natural experiments”), referring to any kind of naturally occurring circumstances in which subsets of the population have different levels of exposure to a supposed causal factor [103]. Currently, a man-made famine, where dietary insults and chronic stress tend to co-occur in exposed populations, is typically used in quasi-experimental design [104]. The nutritional status might be affected by stressful events at different levels including self-selection of dietary components, intake of calories, and utilization of metabolic wastes for energy production, whereas nutritional factors can, in turn, affect stress response through influencing both peripheral and central mechanisms of stress reactivity [14, 105]. Thereby, famine has numerous features that can be beneficial for its use as a natural experiment in studying lasting outcomes of stressful events in early life [104, 106], although using such research design could confound attempts to distinguish the (intergenerational) effects of nutrition and stress.

In a number of quasi-experimental statues, impaired cognitive and behavioral functioning, as well as psychiatric illness in adulthood, has been reported in cohorts exposed in their early life to natural disasters or stressful historic events, such as World War II [107], Holocaust [108–110], Israeli-Arab war of 1967 [111, 112], Chinese Famine of 1959–1961 [113–117], and Dutch famine of 1944–1945 [118–120]. In some recent quasi-experimental studies, evidence for the epigenetic embedding of stressful historic events was obtained. Although no relationship between the exposure to the Dutch famine throughout the intrauterine period and whole-genome DNA methylation level in adulthood was observed [121], an association between the famine exposure during early gestation and changed patterns of methylation at CpG dinucleotides in genes responsible for growth, development, and metabolism in the whole blood of adult individuals was found in the Tobi et al. study [122]. The gene-specific differences in the patterns of DNA methylation associated with in-utero exposure to the Dutch famine have been indicated in several studies including those of Heijmans et al. [123] and Tobi et al. [124]. The differences in DNA methylation levels in HPA axis-associated genes, such as corticotropin-releasing hormone (CRH) and NR3C1 genes, however, were non-significant in the latest study. Importantly, the effect of prenatal famine exposure has been demonstrated to strongly depend on the exposure period, with differences being most pronounced when the famine exposure occurred throughout the periconceptional period rather than throughout the late gestational developmental stage. These findings demonstrate that early intrauterine period is the most sensitive stage in human ontogenesis [125, 126]. Since epigenome is demonstrated to be most plastic during this ontogenetic period, these data are suggestive for the role of epigenetic modifications in driving life-lasting effects of exposures to famine and/or other disasters in early life.

## Transgenerational transmission of childhood trauma

Evidence has been also obtained that early adversity-induced neuronal and behavioral effects can be transgenerationally transmitted via epigenetic mechanisms to subsequent generations [45, 127]. In the study by Yehuda et al. [128], the Holocaust survivor offspring were studied which are known to have altered GR sensitivity and vulnerability to psychiatric disorders. In this study, adult offspring with both maternal and paternal Holocaust-induced posttraumatic stress disorder (PTSD) demonstrated decreased levels of the 1F NR3C1 promoter methylation, while offspring with paternal PTSD only exhibited higher methylation levels of 1F NR3C1 promoter in the peripheral blood mononuclear cells compared to participants without parental Holocaust exposure. Similar transgenerational effects were found for another historical event, such as the Tutsi genocide. In the Perroud et al. study [129], mothers exposed to the Tutsi genocide as well as their offspring had higher mineralocorticoid receptor levels and lower cortisol and GR levels in comparison with non-exposed mothers and their children. Furthermore, the exposed mothers and their progeny had higher levels of methylation of the 1F NR3C1 exon than non-exposed subjects.

To examine the mechanisms underlying such transgenerational effects of paternal trauma, Gapp et al. [130] using a mouse model of unpredictable maternal separation and maternal stress have demonstrated that postnatal trauma changes coping behavior in adverse conditions in exposed males when adult and in their adult male offspring. These behavioral changes have been accompanied by elevated levels of NR3C1 expression and reduced methylation of the NR3C1 promoter in the hippocampus. The DNA methylation levels were also lowered in sperm cells of exposed males when adult. Interestingly, the transgenerational transmission of neurobehavioral symptoms has been shown to be prevented by paternal environmental enrichment, and this effect was linked to the reversal of changes in DNA methylation and expression of NR3C1 gene in the hippocampus of the male offspring.

## Conclusion

Accumulating evidence assume that adversity early in life can be associated with later neuropsychiatric, cognitive, and behavioral outcomes. Recent findings suggest that childhood adversity can have greater impact on the later health status than stressful exposures in adulthood, as assumed by phenomenon of the biological embedding of early experience. In several studies, credible evidence is obtained that adversity early in life can reach far into the later adulthood partly due to cellular aging, as evident from recent data indicating that severe traumatic and social exposures as well as institutional care history in childhood can be embedded at the molecular level through accelerated telomere shortening [131–133].

It is increasingly clear that epigenetic control of gene expression plays a central role in these effects. Some authors hypothesized that such early-life “epigenetic tuning” may likely prepare particular genes for responses to subsequent triggers [134]. By this mechanism, the functional performance of different tissues and organs may be established well before they are actually challenged. In evolutionary terms, such epigenetic fine-tuning of the expression of responsible genes enables the organism to adapt to varying environmental conditions [135], but it can enhance the risk for disorders, including neuropsychiatric ones, later in life [136]. Thus, epigenetic studies can provide insight into the mechanisms mediating the relationship between early-life adversities, aberrant neuroplastic interactions, and adult health outcomes. The role of particular genes such as hippocampal NR3C1 as well as genes coding arginine vasopressin and CRF in the neurons of the paraventricular nucleus has been demonstrated in mediating the effects of early adversity-associated pathological conditions [70, 137].

In the past decade, significant advances have been achieved in the emerging field of neurobehavioral epigenetics. Some research challenges, however, must be addressed for further progress in the field. For example, it is still not clear how stable are modifications of epigenetic marks which are induced by adversities in early life. Recent findings suggest that such epigenetic modifications can be long-term or even life-long and may persist up to the highest age categories [138–140]. These data, however, are rather scarce; therefore, it requires further investigation. Another issue is that epigenetic patterns can be specific not only for distinct cell types but also for specific neuronal pathways in the same brain regions [141]. Thus, focus of further research will likely be shifted from particular candidate genes to particular candidate gene pathways that may be epigenetically labile in response to adverse conditions in early life. Moreover, as significant epigenetic modifications originate both within and among different types of tissues, one more potentially important issue is applicability of samples from peripheral blood for determining epigenetic modifications in human studies. Indeed, though widespread epigenetic changes might be induced by traumatic experiences in early life, these effects may greatly vary in magnitude and direction in neuronal tissues relative to non-neuronal tissues including peripheral blood. This may limit the opportunity to studying the long-term neurobehavioral impacts of early trauma basing only on peripheral blood samples or buccal swabs. Therefore, animal models, which provide an opportunity to simultaneously measure the epigenetic profiles in both peripheral and brain tissues, can be useful in highlighting epigenetic pathways underlying the biological embedding of early adversity. The use of animal models certainly raises questions concerning the specificity of such pathways across different mammalian species and about similarities and differences among these pathways in various animal species and humans. In addition, the potential impact of subsequent exposure to traumatic events during adolescence and adulthood on the process of epigenetic embedding of early experiences also needs to be considered as the findings from some investigations suggest that epigenome continues to be labile during adulthood [142].

A genuine incorporation of novel knowledge about the mechanisms underlying the process of epigenetic embedding of adverse experiences during sensitive developmental periods into the current paradigm on the causation of adult neurobehavioral and cognitive dysfunction will certainly move the focus of efforts targeted toward prevention of adult psychopathological conditions from the later life stages to early developmental stages from conception to weaning. Indeed, reducing or eliminating risk factors early in life would likely have a potential to prevent neurological dysfunctions and psychopathologies in adult life. In this context, an important point is that epigenetic states, in contrast to the relatively stable genetic information, are reversible and can be modified by environmental factors [143]. Therefore, modulation of epigenetic pathways involved in biological embedding may provide promising new direction toward novel therapeutic strategies against neurological and cognitive dysfunctions in adult life.

Over the last years, the therapeutic potential of pharmaceuticals targeted at chromatin modifying enzymes, such as histone deacetylase (HDAC) inhibitors, in treatment of cognitive and behavioral impairments as well as psychiatric disorders such as anxiety, depression, fear, and schizophrenia has been repeatedly demonstrated [144–146]. In a number of preclinical animal models, the convincing evidence is obtained that treatment with HDAC inhibitors can be effective in the prevention and therapy of experimentally induced cognitive and behavioral abnormalities. Treatment with the HDAC inhibitor sodium butyrate (SB) reversed the abnormal hyperactive behavior in a rat model of D-amphetamine-induced mania-like behavior [147]. Furthermore, SB and other HDAC inhibitor, valproate, abolished manic-like behaviors and protected the rat brain from metabolic disturbances induced by the metabolic poison, ouabain [148]. These findings were subsequently confirmed in models of depressive- and manic-like behaviors induced by chronic mild stress or maternal deprivation [149]. In the same models, SB treatment improved the recognition memory and reversed the stress-induced decrease of hippocampal neurotrophic factors including BDNF, nerve growth factor, and glial cell line-derived neurotrophic factor [150], and also abolished the maternal deprivation- and chronic mild stress-induced dysfunction in the striatum of rats [151]. In a mouse model of valproic acid (VPA)-induced autism, chronicadministration of SB resulted in attenuating the experimentally induced deficits in a novel object recognition and loss of hippocampal dendritic spine, as well as significantly increased level of acetylation of the histone H3 in hippocampus [152]. In the same mouse model of VPA-induced autism, SB attenuated autism-like deficits in social behavior and modified transcription levels of many behavior-associated genes in the prefrontal cortex, in particular, genes implicated in neuronal excitation or inhibition [153]. In a mouse model of isoflurane-induced cognitive deficits, treatment with SB attenuated the repression of contextual fear memory, apparently by promoting histone acetylation and expression of histone acetylation-mediated genes [154]. In the 6-hydroxydopamine-induced Parkinson’s disease rat model, administration of SB resulted in a substantial attenuation of motor deficits and also in an increase of striatal dopamine, BDNF, and global H3 histone acetylation levels [155]. The cognition-protective effect of SB was revealed in a rat model of chronic cerebral hypoperfusion; this effect was, at least in part, mediated through enhancing histone acetylation and facilitating the transcription of Nrf2 downstream genes in the hippocampus [156]. HDAC inhibitor phenylbutyrate (PBA) was shown to be able to attenuate hippocampal neuronal loss and reverse the Alzheimer’s disease-like phenotype in a mouse model of Alzheimer’s disease [157]. In rats with neonatal ventral hippocampal lesions which are commonly used for modeling neurodevelopmental aspects of schizophrenia, treatment with PBA reversed the unfavorable behavioral consequences of these lesions in the ventral hippocampus [158]. In a maternal separation rat model, treatment with another HDAC inhibitor, suberoylanilide hydroxamic acid (SAHA), reversed early-life stress-induced visceral hypersensitivity and anxiety behavior [159]. Taken together, these results suggest that targeting epigenome by specific pharmacological interventions can be a promising therapeutic option in treatment of neuropsychiatric and cognitive impairments, including those related to biological embedding of early life exposures. There are certainly many important issues which need to be addressed before implementation of such interventions in clinical practice, including their effective dose levels, administration frequency, safety, and potential side effects. These issues remain to be addressed in future clinical trials.

## Abbreviations

BDNF: Brain-derived neurotrophic factor
CRF: Hypothalamic corticotropin-releasing factor
CRH: Corticotropin-releasing hormone
EWAS: Epigenome-wide association studies
GR: Glucocorticoid receptor
HDAC: Histone deacetylase
HPA: Hypothalamic-pituitary-adrenal
LG: Licking/grooming
MAOA: Monoamine oxidase A
MWAS: Methylome-wide association study
NR3C1: Nuclear receptor subfamily 3 group C member
PBA: Phenylbutyrate
PTSD: Posttraumatic stress disorder
SAHA: Suberoylanilide hydroxamic acid
SB: Sodium butyrate
SES: Socioeconomic status
VPA: Valproic acid

## References

[1] Hanson MA, Gluckman PD (2015). Developmental origins of health and disease—global public health implications. Best Pract Res Clin Obstet Gynaecol.

[2] Eriksson JG (2016). Developmental origins of health and disease—from a small body size at birth to epigenetics. Ann Med.

[3] Champagne FA (2012). Interplay between social experiences and the genome: epigenetic consequences for behavior. Adv Genet.

[4] Doherty TS, Roth TL (2016). Insight from animal models of environmentally driven epigenetic changes in the developing and adult brain. Dev Psychopathol.

[5] Curley JP, Champagne FA (2016). Influence of maternal care on the developing brain: mechanisms, temporal dynamics and sensitive periods. Front Neuroendocrinol.

[6] Kim DR, Bale TL, Epperson CN (2015). Prenatal programming of mental illness: current understanding of relationship and mechanisms. Curr Psychiatry Rep.

[7] Fowden AL, Valenzuela OA, Vaughan OR, Jellyman JK, Forhead AJ (2016). Glucocorticoid programming of intrauterine development. Domest Anim Endocrinol.

[8] Silberman DM, Acosta GB, Zorrilla Zubilete MA (2016). Long-term effects of early life stress exposure: role of epigenetic mechanisms. Pharmacol Res.

[9] Maccari S, Polese D, Reynaert ML, Amici T, Morley-Fletcher S, Fagioli F (2017). Early-life experiences and the development of adult diseases with a focus on mental illness: the human birth theory. Neuroscience.

[10] Weinstock M (2008). The long-term behavioural consequences of prenatal stress. Neurosci Biobehav Rev.

[11] Reynolds RM, Jacobsen GH, Drake AJ (2013). What is the evidence in humans that DNA methylation changes link events in utero and later life disease?. Clin Endocrinol.

[12] Reynolds RM, Labad J, Buss C, Ghaemmaghami P, Räikkönen K (2013). Transmitting biological effects of stress in utero: implications for mother and offspring. Psychoneuroendocrinology.

[13] Barbazanges A, Piazza PV, Le Moal M, Maccari S (1996). Maternal glucocorticoid secretion mediates long-term effects of prenatal stress. J Neurosci.

[14] Entringer S, Buss C, Wadhwa PD (2010). Prenatal stress and developmental programming of human health and disease risk: concepts and integration of empirical findings. Curr Opin Endocrinol Diabetes Obes.

[15] Entringer S, Buss C, Wadhwa PD (2015). Prenatal stress, development, health and disease risk: a psychobiological perspective—2015 Curt Richter Award Paper. Psychoneuroendocrinology.

[16] Lippmann M, Bress A, Nemeroff CB, Plotsky PM, Monteggia LM (2007). Long-term behavioural and molecular alterations associated with maternal separation in rats. Eur J Neurosci.

[17] Nishi M, Horii-Hayashi N, Sasagawa T (2014). Effects of early life adverse experiences on the brain: implications from maternal separation models in rodents. Front Neurosci.

[18] Tractenberg SG, Levandowski ML, de Azeredo LA, Orso R, Roithmann LG, Hoffmann ES (2016). An overview of maternal separation effects on behavioural outcomes in mice: evidence from a four-stage methodological systematic review. Neurosci Biobehav Rev.

[19] Braun K, Champagne FA (2014). Paternal influences on offspring development: behavioural and epigenetic pathways. J Neuroendocrinol.

[20] Korosi A, Baram TZ (2010). Plasticity of the stress response early in life: mechanisms and significance. Dev Psychobiol.

[21] Perry R, Sullivan RM (2014). Neurobiology of attachment to an abusive caregiver: short-term benefits and long-term costs. Dev Psychobiol.

[22] Szyf M, Tang YY, Hill KG, Musci R (2016). The dynamic epigenome and its implications for behavioral interventions: a role for epigenetics to inform disorder prevention and health promotion. Transl Behav Med.

[23] Cowan CS, Callaghan BL, Kan JM, Richardson R (2016). The lasting impact of early-life adversity on individuals and their descendants: potential mechanisms and hope for intervention. Genes Brain Behav..

[24] Canovas S, Ross PJ (2016). Epigenetics in preimplantation mammalian development. Theriogenology.

[25] Vaiserman A (2015). Epidemiologic evidence for association between adverse environmental exposures in early life and epigenetic variation: a potential link to disease susceptibility?. Clin Epigenetics.

[26] Vaiserman AM, Koliada AK, Jirtle RL (2017). Non-genomic transmission of longevity between generations: potential mechanisms and evidence across species. Epigenetics Chromatin.

[27] Lee HJ, Hore TA, Reik W (2014). Reprogramming the methylome: erasing memory and creating diversity. Cell Stem Cell.

[28] Trerotola M, Relli V, Simeone P, Alberti S (2015). Epigenetic inheritance and the missing heritability. Hum Genomics.

[29] Vickaryous N, Whitelaw E (2005). The role of the early embryonic environment on epigenotype and phenotype. Reprod Fertil Dev.

[30] Hochberg Z, Feil R, Constancia M, Fraga M, Junien C, Carel JC (2011). Child health, developmental plasticity, and epigenetic programming. Endocr Rev.

[31] Feil R, Fraga MF (2012). Epigenetics and the environment: emerging patterns and implications. Nat Rev Genet..

[32] Boyce WT, Kobor MS (2015). Development and the epigenome: the ‘synapse’ of gene–environment interplay. Dev Sci.

[33] McGowan PO, Roth TL (2015). Epigenetic pathways through which experiences become linked with biology. Dev Psychopathol.

[34] Provencal N, Binder EB (2015). The neurobiological effects of stress as contributors to psychiatric disorders: focus on epigenetics. Curr Opin Neurobiol.

[35] Isles AR (2015). Neural and behavioral epigenetics; what it is, and what is hype. Genes Brain Behav.

[36] Halldorsdottir T, Binder EB (2017). Gene × environment interactions: from molecular mechanisms to behavior. Annu Rev Psychol.

[37] Mueller BR, Bale TL (2008). Sex-specific programming of offspring emotionality after stress early in pregnancy. J Neurosci.

[38] O’Donnell D, Larocque S, Seckl JR, Meaney MJ (1994). Postnatal handling alters glucocorticoid, but not mineralocorticoid messenger RNA expression in the hippocampus of adult rats. Brain Res Mol Brain Res.

[39] Weaver IC, Cervoni N, Champagne FA, D'Alessio AC, Sharma S, Seckl JR (2004). Epigenetic programming by maternal behavior. Nat Neurosci.

[40] Kundakovic M, Gudsnuk K, Herbstman JB, Tang D, Perera FP, Champagne FA (2015). DNA methylation of BDNF as a biomarker of early-life adversity. Proc Natl Acad Sci U S A.

[41] Monk C, Spicer J, Champagne FA (2012). Linking prenatal maternal adversity to developmental outcomes in infants: the role of epigenetic pathways. Dev Psychopathol.

[42] Lewis AJ, Austin E, Knapp R, Vaiano T, Galbally M (2015). Perinatal maternal mental health, fetal programming and child development. Healthcare (Basel).

[43] Newman L, Judd F, Olsson CA, Castle D, Bousman C, Sheehan P (2016). Early origins of mental disorder—risk factors in the perinatal and infant period. BMC Psychiatry.

[44] Babenko O, Kovalchuk I, Metz GA (2015). Stress-induced perinatal and transgenerational epigenetic programming of brain development and mental health. Neurosci Biobehav Rev.

[45] Nemoda Z, Massart R, Suderman M, Hallett M, Li T, Coote M (2015). Maternal depression is associated with DNA methylation changes in cord blood T lymphocytes and adult hippocampi. Transl Psychiatry.

[46] Braithwaite EC, Kundakovic M, Ramchandani PG, Murphy SE, Champagne FA (2015). Maternal prenatal depressive symptoms predict infant NR3C1 1F and BDNF IV DNA methylation. Epigenetics.

[47] Oberlander TF, Weinberg J, Papsdorf M, Grunau R, Misri S, Devlin AM (2008). Prenatal exposure to maternal depression, neonatal methylation of human glucocorticoid receptor gene (NR3C1) and infant cortisol stress responses. Epigenetics.

[48] Radtke KM, Ruf M, Gunter HM, Dohrmann K, Schauer M, Meyer A (2011). Transgenerational impact of intimate partner violence on methylation in the promoter of the glucocorticoid receptor. Transl Psychiatry.

[49] Hompes T, Izzi B, Gellens E, Morreels M, Fieuws S, Pexsters A (2013). Investigating the influence of maternal cortisol and emotional state during pregnancy on the DNA methylation status of the glucocorticoid receptor gene (NR3C1) promoter region in cord blood. J Psychiatr Res.

[50] Provenzi L, Giorda R, Beri S, Montirosso R (2016). SLC6A4 methylation as an epigenetic marker of life adversity exposures in humans: a systematic review of literature. Neurosci Biobehav Rev.

[51] Devlin AM, Brain U, Austin J, Oberlander TF (2010). Prenatal exposure to maternal depressed mood and the MTHFR C677T variant affect SLC6A4 methylation in infants at birth. PLoS One.

[52] Conradt E, Lester BM, Appleton AA, Armstrong DA, Marsit CJ (2013). The roles of DNA methylation of NR3C1 and 11β–HSD2 and exposure to maternal mood disorder in utero on newborn neurobehavior. Epigenetics.

[53] Ponder KL, Salisbury A, McGonnigal B, Laliberte A, Lester B, Padbury JF (2011). Maternal depression and anxiety are associated with altered gene expression in the human placenta without modification by antidepressant use: implications for fetal programming. Dev Psychobiol.

[54] O’Donnell KJ, Bugge Jensen A, Freeman L, Khalife N, O'Connor TG, Glover V (2012). Maternal prenatal anxiety and downregulation of placental 11β–HSD2. Psychoneuroendocrinology.

[55] Appleton AA, Armstrong DA, Lesseur C, Lee J, Padbury JF, Lester BM (2013). Patterning in placental 11–B hydroxysteroid dehydrogenase methylation according to prenatal socioeconomic adversity. PLoS One.

[56] Bale TL (2015). Epigenetic and transgenerational reprogramming of brain development. Nat Rev Neurosci.

[57] Qiao Y, Yang X, Jing N (2016). Epigenetic regulation of early neural fate commitment. Cell Mol Life Sci.

[58] Varese F, Smeets F, Drukker M, Lieverse R, Lataster T, Viechtbauer W (2012). Childhood adversities increase the risk of psychosis: a meta-analysis of patient-control, prospective- and cross-sectional cohort studies. Schizophr Bull.

[59] Brent DA, Silverstein M (2013). Shedding light on the long shadow of childhood adversity. JAMA.

[60] Strüber N, Strüber D, Roth G (2014). Impact of early adversity on glucocorticoid regulation and later mental disorders. Neurosci Biobehav Rev.

[61] Chiang JJ, Taylor SE, Bower JE (2015). Early adversity, neural development, and inflammation. Dev Psychobiol.

[62] Ehrlich KB, Ross KM, Chen E, Miller GE (2016). Testing the biological embedding hypothesis: is early life adversity associated with a later proinflammatory phenotype?. Dev Psychopathol.

[63] Danese A, McEwen BS (2012). Adverse childhood experiences, allostasis, allostatic load, and age-related disease. Physiol Behav.

[64] Remmes J, Bodden C, Richter SH, Lesting J, Sachser N, Pape HC (2016). Impact of life history on fear memory and extinction. Front Behav Neurosci.

[65] Rubin LP (2016). Maternal and pediatric health and disease: integrating biopsychosocial models and epigenetics. Pediatr Res.

[66] Meaney MJ, Szyf M, Seckl JR (2007). Epigenetic mechanisms of perinatal programming of hypothalamic–pituitary–adrenal function and health. Trends Mol Med.

[67] Hackman DA, Farah MJ, Meaney MJ (2010). Socioeconomic status and the brain: mechanistic insights from human and animal research. Nat Rev Neurosci.

[68] Maccari S, Krugers HJ, Morley-Fletcher S, Szyf M, Brunton PJ (2014). The consequences of early-life adversity: neurobiological, behavioural and epigenetic adaptations. J Neuroendocrinol.

[69] Bick J, Naumova O, Hunter S, Barbot B, Lee M, Luthar SS (2012). Childhood adversity and DNA methylation of genes involved in the hypothalamus–pituitary–adrenal axis and immune system: whole–genome and candidate–gene associations. Dev Psychopathol.

[70] Vialou V, Feng J, Robison AJ, Nestler EJ (2013). Epigenetic mechanisms of depression and antidepressant action. Annu Rev Pharmacol Toxicol.

[71] Smart C, Strathdee G, Watson S, Murgatroyd C, McAllister-Williams RH (2015). Early life trauma, depression and the glucocorticoid receptor gene––an epigenetic perspective. Psychol Med.

[72] Turecki G, Meaney MJ (2016). Effects of the social environment and stress on glucocorticoid receptor gene methylation: a systematic review. Biol Psychiatry.

[73] DeLisi M, Vaughn MG (2015). The vindication of Lamarck? Epigenetics at the intersection of law and mental health. Behav Sci Law.

[74] Frodl T (2017). Do (epi)genetics impact the brain in functional neurologic disorders?. Handb Clin Neurol.

[75] Heim C, Newport DJ, Mletzko T, Miller AH, Nemeroff CB (2008). The link between childhood trauma and depression: insights from HPA axis studies in humans. Psychoneuroendocrinology.

[76] Heim C, Binder EB (2012). Current research trends in early life stress and depression: review of human studies on sensitive periods, gene–environment interactions, and epigenetics. Exp Neurol.

[77] Heim C, Newport DJ, Heit S, Graham YP, Wilcox M, Bonsall R (2000). Pituitary–adrenal and autonomic responses to stress in women after sexual and physical abuse in childhood. JAMA.

[78] Frazzetto G, Di Lorenzo G, Carola V, Proietti L, Sokolowska E, Siracusano A (2007). Early trauma and increased risk for physical aggression during adulthood: the moderating role of MAOA genotype. PLoS One.

[79] Byrd AL, Manuck SB (2014). MAOA, childhood maltreatment, and antisocial behavior: meta-analysis of a gene–environment interaction. Biol Psychiatry.

[80] Borghol N, Suderman M, McArdle W, Racine A, Hallett M, Pembrey M (2012). Associations with early-life socio-economic position in adult DNA methylation. Int J Epidemiol.

[81] Miller GE, Chen E, Fok AK, Walker H, Lim A, Nicholls EF (2009). Low early-life social class leaves a biological residue manifested by decreased glucocorticoid and increased proinflammatory signaling. Proc Natl Acad Sci U S A.

[82] Chen EE, Miller GE, Kobor MS, Cole SW (2011). Maternal warmth buffers the effects of low early-life socioeconomic status on pro-inflammatory signaling in adulthood. Mol Psychiatry.

[83] Hornung OP, Heim CM (2014). Gene–environment interactions and intermediate phenotypes: early trauma and depression. Front Endocrinol (Lausanne).

[84] Labonté B, Suderman M, Maussion G, Navaro L, Yerko V, Mahar I (2012). Genome-wide epigenetic regulation by early-life trauma. Arch Gen Psychiatry.

[85] Suderman M, Borghol N, Pappas JJ, Pinto Pereira SM, Pembrey M, Hertzman C (2014). Childhood abuse is associated with methylation of multiple loci in adult DNA. BMC Med Genet.

[86] Provençal N, Suderman MJ, Guillemin C, Vitaro F, Côté SM, Hallett M (2014). Association of childhood chronic physical aggression with a DNA methylation signature in adult human T cells. PLoS One.

[87] Julian MM (2013). Age at adoption from institutional care as a window into the lasting effects of early experiences. Clin Child Fam Psychol Rev.

[88] Naumova O, Lee M, Koposov R, Szyf M, Dozier M, Grigorenko EL (2012). Differential patterns of whole-genome DNA methylation in institutionalized children and children raised by their biological parents. Dev Psychopathol.

[89] McGowan PO, Sasaki A, Huang TC, Unterberger A, Suderman M, Ernst C (2008). Promoter-wide hypermethylation of the ribosomal RNA gene promoter in the suicide brain. PLoS One.

[90] McGowan PO, Sasaki A, D’Alessio AC, Dymov S, Labonté B, Szyf M (2009). Epigenetic regulation of the glucocorticoid receptor in human brain associates with childhood abuse. Nature Neurosci.

[91] Labonté B, Yerko V, Gross J, Mechawar N, Meaney MJ, Szyf M (2012). Differential glucocorticoid receptor exon 1(B), 1(C), and 1(H) expression and methylation in suicide completers with a history of childhood abuse. Biol Psychiatry.

[92] Bustamante AC, Aiello AE, Galea S, Ratanatharathorn A, Noronha C, Wildman DE (2016). Glucocorticoid receptor DNA methylation, childhood maltreatment and major depression. J Affect Disord.

[93] Tyrka AR, Ridout KK, Parade SH (2016). Childhood adversity and epigenetic regulation of glucocorticoid signaling genes: associations in children and adults. Dev Psychopathol.

[94] Tyrka AR, Parade SH, Welch ES, Ridout KK, Price LH, Marsit C (2016). Methylation of the leukocyte glucocorticoid receptor gene promoter in adults: associations with early adversity and depressive, anxiety and substance-use disorders. Transl Psychiatry.

[95] Klengel T, Mehta D, Anacker C, Rex-Haffner M, Pruessner JC, Pariante CM, Pace TW (2013). Allele-specific FKBP5 DNA demethylation mediates gene-childhood trauma interactions. Nat Neurosci.

[96] Tyrka AR, Price LH, Marsit C, Walters OC, Carpenter LL (2012). Childhood adversity and epigenetic modulation of the leukocyte glucocorticoid receptor: preliminary findings in healthy adults. PLoS One.

[97] Perroud N, Paoloni-Giacobino A, Prada P, Olié E, Salzmann A, Nicastro R (2011). Increased methylation of glucocorticoid receptor gene (NR3C1) in adults with a history of childhood maltreatment: a link with the severity and type of trauma. Transl Psychiatry.

[98] Steiger H, Labonté B, Groleau P, Turecki G, Israel M (2013). Methylation of the glucocorticoid receptor gene promoter in bulimic women: associations with borderline personality disorder, suicidality, and exposure to childhood abuse. Int J Eat Disord.

[99] Booij L, Szyf M, Carballedo A, Frey EM, Morris D, Dymov S (2015). DNA methylation of the serotonin transporter gene in peripheral cells and stress-related changes in hippocampal volume: a study in depressed patients and healthy controls. PLoS One.

[100] Miller G, Chen E (2007). Unfavorable socioeconomic conditions in early life presage expression of proinflammatory phenotype in adolescence. Psychosom Med.

[101] Witek-Janusek L, Tell D, Gaylord-Harden N, Mathews HL (2017). Relationship of childhood adversity and neighborhood violence to a proinflammatory phenotype in emerging adult African American men: an epigenetic link. Brain Behav Immun.

[102] Davidson RJ, McEwen BS (2012). Social influences on neuroplasticity: stress and interventions to promote well-being. Nat Neurosci.

[103] Vaiserman A (2011). Early-life origin of adult disease: evidence from natural experiments. Exp Gerontol.

[104] Mill J, Heijmans BT (2013). From promises to practical strategies in epigenetic epidemiology. Nat Rev Genet.

[105] Entringer S, Wadhwa PD (2013). Developmental programming of obesity and metabolic dysfunction: role of prenatal stress and stress biology. Nestle Nutr Inst Workshop Ser.

[106] Steiger H, Thaler L (2016). Eating disorders, gene–environment interactions and the epigenome: roles of stress exposures and nutritional status. Physiol Behav.

[107] Kesternich I, Siflinger B, Smith JP, Winter JK (2014). The effects of World War II on economic and health outcomes across Europe. Rev Econ Stat.

[108] Yehuda R, Bierer LM (2008). Transgenerational transmission of cortisol and PTSD risk. Prog Brain Res.

[109] Bercovich E, Keinan-Boker L, Shasha SM (2014). Long-term health effects in adults born during the Holocaust. Isr Med Assoc J.

[110] Keinan-Boker L, Shasha-Lavsky H, Eilat-Zanani S, Edri-Shur A, Shasha SM (2015). Chronic health conditions in Jewish Holocaust survivors born during World War II. Isr Med Assoc J.

[111] Malaspina D, Corcoran C, Kleinhaus KR, Perrin MC, Fennig S, Nahon D (2008). Acute maternal stress in pregnancy and schizophrenia in offspring: a cohort prospective study. BMC Psychiatry.

[112] Kleinhaus K, Harlap S, Perrin M, Manor O, Margalit-Calderon R, Opler M (2013). Prenatal stress and affective disorders in a population birth cohort. Bipolar Disord.

[113] St Clair D, Xu M, Wang P, Yu Y, Fang Y, Zhang F (2005). Rates of adult schizophrenia following prenatal exposure to the Chinese famine of 1959–1961. JAMA.

[114] MQ X, Sun WS, Liu BX, Feng GY, Yu L, Yang L (2009). Prenatal malnutrition and adult schizophrenia: further evidence from the 1959–1961 Chinese famine. Schizophr Bull.

[115] Song S, Wang W, Hu P (2009). Famine, death, and madness: schizophrenia in early adulthood after prenatal exposure to the Chinese Great Leap Forward Famine. Soc Sci Med.

[116] Huang C, Phillips MR, Zhang Y, Zhang J, Shi Q, Song Z (2013). Malnutrition in early life and adult mental health: evidence from a natural experiment. Soc Sci Med.

[117] Wang C, An Y, Yu H, Feng L, Liu Q, Lu Y (2016). Association between exposure to the Chinese famine in different stages of early life and decline in cognitive functioning in adulthood. Front Behav Neurosci.

[118] de Rooij SR, Painter RC, Phillips DI, Osmond C, Tanck MW, Bossuyt PM (2006). Cortisol responses to psychological stress in adults after prenatal exposure to the Dutch famine. Psychoneuroendocrinology.

[119] de Rooij SR, Veenendaal MV, Räikkönen K, Roseboom TJ (2012). Personality and stress appraisal in adults prenatally exposed to the Dutch famine. Early Hum Dev.

[120] Susser E, St Clair D (2013). Prenatal famine and adult mental illness: interpreting concordant and discordant results from the Dutch and Chinese Famines. Soc Sci Med.

[121] Lumey LH, Terry MB, Delgado-Cruzata L, Liao Y, Wang Q, Susser E (2012). Adult global DNA methylation in relation to pre-natal nutrition. Int J Epidemiol.

[122] Tobi EW, Slieker RC, Stein AD, Suchiman HE, Slagboom PE, van Zwet EW (2015). Early gestation as the critical time-window for changes in the prenatal environment to affect the adult human blood methylome. Int J Epidemiol.

[123] Heijmans BT, Tobi EW, Stein AD, Putter H, Blauw GJ, Susser ES (2008). Persistent epigenetic differences associated with prenatal exposure to famine in humans. Proc Natl Acad Sci U S A.

[124] Tobi EW, Lumey LH, Talens RP, Kremer D, Putter H, Stein AD (2009). DNA methylation differences after exposure to prenatal famine are common and timing- and sex-specific. Hum Mol Genet.

[125] Heijmans BT, Tobi EW, Lumey LH, Slagboom PE (2009). The epigenome: archive of the prenatal environment. Epigenetics.

[126] Roseboom TJ, Painter RC, van Abeelen AF, Veenendaal MV, de Rooij SR (2011). Hungry in the womb: what are the consequences? Lessons from the Dutch famine. Maturitas.

[127] Gröger N, Matas E, Gos T, Lesse A, Poeggel G, Braun K (2016). The transgenerational transmission of childhood adversity: behavioral, cellular, and epigenetic correlates. J Neural Transm (Vienna).

[128] Yehuda R, Daskalakis NP, Lehrner A, Desarnaud F, Bader HN, Makotkine I (2014). Influences of maternal and paternal PTSD on epigenetic regulation of the glucocorticoid receptor gene in Holocaust survivor offspring. Am J Psychiatry.

[129] Perroud N, Rutembesa E, Paoloni-Giacobino A, Mutabaruka J, Mutesa L, Stenz L (2014). The Tutsi genocide and transgenerational transmission of maternal stress: epigenetics and biology of the HPA axis. World J Biol Psychiatry.

[130] Gapp K, Bohacek J, Grossmann J, Brunner AM, Manuella F, Nanni P (2016). Potential of environmental enrichment to prevent transgenerational effects of paternal trauma. Neuropsychopharmacology.

[131] Puterman E, Gemmill A, Karasek D, Weir D, Adler N.E, Prather AA, et al. Lifespan adversity and later adulthood telomere length in the nationally representative US Health and Retirement Study. Proc Natl Acad Sci U S A 2016;113(42):E6335–E6342. 10.1073/pnas.1525602113 PMC5081642 27698131

[132] Humphreys KL, Esteves K, Zeanah CH, Fox NA, Nelson CA (2016). Accelerated telomere shortening: tracking the lasting impact of early institutional care at the cellular level. Psychiatry Res.

[133] Mitchell C, Hobcraft J, McLanahan SS, Siegel SR, Berg A, Brooks-Gunn J (2014). Social disadvantage, genetic sensitivity, and children’s telomere length. Proc Natl Acad Sci U S A.

[134] Scott BR, Belinsky SA, Leng S, Lin Y, Wilder JA, Damiani LA (2009). Radiation-stimulated epigenetic reprogramming of adaptive-response genes in the lung: an evolutionary gift for mounting adaptive protection against lung cancer. Dose-Response.

[135] Barouki R, Gluckman PD, Grandjean P, Hanson M, Heindel JJ (2012). Developmental origins of non-communicable disease: implications for research and public health. Environ Health.

[136] Godfrey KM, Costello PM, Lillycrop KA (2015). The developmental environment, epigenetic biomarkers and long-term health. J Dev Orig Health Dis.

[137] Palma-Gudiel H, Córdova-Palomera A, Leza JC, Fañanás L (2015). Glucocorticoid receptor gene (NR3C1) methylation processes as mediators of early adversity in stress-related disorders causality: a critical review. Neurosci Biobehav Rev.

[138] Turecki G, Ota VK, Belangero SI, Jackowski A, Kaufman J (2014). Early life adversity, genomic plasticity, and psychopathology. Lancet Psychiatry.

[139] Vaiserman AM (2014). Early-life nutritional programming of longevity. J Dev Orig Health Dis.

[140] Kundakovic M, Jaric I (2017). The epigenetic link between prenatal adverse environments and neurodevelopmental disorders. Genes (Basel).

[141] McGowan PO (2012). Epigenetic clues to the biological embedding of early life adversity. Biol Psychiatry.

[142] Talens RP, Christensen K, Putter H, Willemsen G, Christiansen L, Kremer D (2012). Epigenetic variation during the adult lifespan: cross-sectional and longitudinal data on monozygotic twin pairs. Aging Cell.

[143] Sen P, Shah PP, Nativio R, Berger SL (2016). Epigenetic mechanisms of longevity and aging. Cell.

[144] Penney J, Tsai LH (2014). Histone deacetylases in memory and cognition. Sci Signal.

[145] Fuchikami M, Yamamoto S, Morinobu S, Okada S, Yamawaki Y, Yamawaki S (2016). The potential use of histone deacetylase inhibitors in the treatment of depression. Prog Neuro-Psychopharmacol Biol Psychiatry.

[146] Qiu X, Xiao X, Li N, Li Y (2017). Histone deacetylases inhibitors (HDACis) as novel therapeutic application in various clinical diseases. Prog Neuro-Psychopharmacol Biol Psychiatry.

[147] Moretti M, Valvassori SS, Varela RB, Ferreira CL, Rochi N, Benedet J (2011). Behavioral and neurochemical effects of sodium butyrate in an animal model of mania. Behav Pharmacol.

[148] Lopes-Borges J, Valvassori SS, Varela RB, Tonin PT, Vieira JS, Gonçalves CL, et al. Histone deacetylase inhibitors reverse manic-like behaviors and protect the rat brain from energetic metabolic alterations induced by ouabain. Pharmacol Biochem Behav. 2015, 128:89–95. 10.1016/j.pbb.2014.11.014 25433326

[149] Resende WR, Valvassori SS, Réus GZ, Varela RB, Arent CO, Ribeiro KF (2013). Effects of sodium butyrate in animal models of mania and depression: implications as a new mood stabilizer. Behav Pharmacol.

[150] Valvassori SS, Varela RB, Arent CO, Dal-Pont GC, Bobsin TS, Budni J (2014). Sodium butyrate functions as an antidepressant and improves cognition with enhanced neurotrophic expression in models of maternal deprivation and chronic mild stress. Curr Neurovasc Res.

[151] Valvassori SS, Resende WR, Budni J, Dal-Pont GC, Bavaresco DV, Réus GZ (2015). Sodium butyrate, a histone deacetylase inhibitor, reverses behavioral and mitochondrial alterations in animal models of depression induced by early- or late-life stress. Curr Neurovasc Res.

[152] Takuma K, Hara Y, Kataoka S, Kawanai T, Maeda Y, Watanabe R (2014). Chronic treatment with valproic acid or sodium butyrate attenuates novel object recognition deficits and hippocampal dendritic spine loss in a mouse model of autism. Pharmacol Biochem Behav.

[153] Kratsman N, Getselter D, Elliott E (2016). Sodium butyrate attenuates social behavior deficits and modifies the transcription of inhibitory/excitatory genes in the frontal cortex of an autism model. Neuropharmacology.

[154] Zhong T, Qing QJ, Yang Y, Zou WY, Ye Z, Yan JQ (2014). Repression of contexual fear memory induced by isoflurane is accompanied by reduction in histone acetylation and rescued by sodium butyrate. Br J Anaesth.

[155] Sharma S, Taliyan R, Singh S (2015). Beneficial effects of sodium butyrate in 6–OHDA induced neurotoxicity and behavioral abnormalities: modulation of histone deacetylase activity. Behav Brain Res.

[156] Liu H, Zhang JJ, Li X, Yang Y, Xie XF, Hu K (2015). Post-occlusion administration of sodium butyrate attenuates cognitive impairment in a rat model of chronic cerebral hypoperfusion. Pharmacol Biochem Behav.

[157] Cuadrado-Tejedor M, Ricobaraza AL, Torrijo R, Franco R, Garcia-Osta A (2013). Phenylbutyrate is a multifaceted drug that exerts neuroprotective effects and reverses the Alzheimer’s disease-like phenotype of a commonly used mouse model. Curr Pharm Des.

[158] Sandner G, Host L, Angst MJ, Guiberteau T, Guignard B, Zwiller J (2011). The HDAC inhibitor phenylbutyrate reverses effects of neonatal ventral hippocampal lesion in rats. Front Psychiatry.

[159] Moloney RD, Stilling RM, Dinan TG, Cryan JF (2015). Early-life stress-induced visceral hypersensitivity and anxiety behavior is reversed by histone deacetylase inhibition. Neurogastroenterol Motil.

